# Consumption of antidiabetic medicines in Portugal: results of a temporal data analysis of a thirteen‐year study (2005–2017)

**DOI:** 10.1186/s12902-021-00686-w

**Published:** 2021-02-24

**Authors:** Artur Mendes Moura, Sofia Oliveira Martins, João Filipe Raposo

**Affiliations:** 1 Faculty of Pharmacy, University of Lisbon, Avenida Professor Gama Pinto, 1649-003 Lisboa, Portugal; 2 Nova Medical School, New University of Lisbon, Lisboa, Portugal; 3 Portugal and Portuguese Diabetes Association (APDP), Lisboa, Portugal

**Keywords:** Diabetes *Mellitus*, Insulin, Hypoglycemic agents, Drug utilization, Portugal

## Abstract

**Background:**

Studies of drug utilization in patients with diabetes, a chronic disease that can be treated with a wide range of available medicines, have attracted substantial social and clinical interest.

**Objective:**

To characterize antidiabetic medicine consumption between 2005 and 2017, to evaluate the trends of these medicines in mainland Portugal, and to compare district consumption. An additional objective was to perform a statistical analysis on drug consumption in different regions of Portugal.

**Methods:**

A descriptive, longitudinal observational study; the setting was mainland Portugal ( excluding Azores and Madeira).

Each medicine has a respective defined daily dose (DDD). The sum of the DDD, provides the annual consumption in terms of the DDD for each district each year. When calculating the annual average for the resident district population and the number of days in a year, the denominator is expressed as 1000 inhabitants per day (TID).

Main outcome measure: The DDD/TID for mainland Portugal (for all districts) between 2005 and 2017 for antidiabetic medicines.

Information was obtained from the official database of prescription medicine invoices with reimbursement in mainland Portugal.

**Results:**

In mainland Portugal, the antidiabetic medicine consumption was 49.3 DDD/TID in 2005 and 88.2 DDD/TID in 2017. The consumption of insulins and their analogs increased from 10.8% to 17.4% compared to the total consumption of antidiabetic medicines.

In 2017, the level of biguanide consumption was 23.1 DDD/TID, that of sulphonylurea consumption was 15.8 DDD/TID, that of DPP-4 inhibitor consumption was 6.8 DDD/TID, and that of SGLT2 inhibitor consumption was 3.0 DDD/TID. The oral consumption of fixed-dose combinations reached 21.4 DDD/TID.

After employing a geographical division between north and south and between coastal and inland regions, the consumption of several different drugs showed statistically significant differences.

**Conclusions:**

When comparing 2017 with 2005, the panorama was quite different, with higher levels of consumption of antidiabetic medicines, insulins and their analogs, noninsulin medicines, long-acting and fast-acting insulins and their analogs, metformin, DPP-4 inhibitors and, mainly, metformin combined with a DPP-4 inhibitor. The SGLT2 inhibitors achieved a representative consumption.

Different consumption patterns may be related to sociodemographic factors or to clinical practices.

## Background

In 1977, the World Health Organization (WHO) defined the concept of drug utilization as the marketing, distribution, prescription, and use of drugs in a society [1]. Drug utilization has medical, social, and economic consequences. In 2003, the WHO published guidelines on drug utilization research, and in addition to their usefulness, highlighted the importance of these studies [2].

Numerous drug utilization studies examining the methodological aspects have been published [3–9]. In this context and as diabetes is a chronic disease, diabetes is appropriate for the application of this type of study. However, few studies have been published in this field.

The high level of prevalence is verified based on the information released by the Organization for Economic Cooperation and Development (OECD) that highlights an age-standardized diabetes prevalence of 6.0 % for the population aged 18–99 years old in 28 European countries and 9.9 % for Portugal using data collected in 2017 [10]. The Portuguese National Diabetes Observatory (*OND*) report showed a prevalence of diabetes of 13.3 % for 2015 in the 20-79-year-old population, which includes an estimated prevalence of 5.8 % for undiagnosed individuals in the same population [11]. We should bear in mind that diabetes prevalence data includes diabetes *mellitus* type 1 and 2, with diabetes *mellitus* 1 representing 5–10 % of existing cases and diabetes *mellitus* type 2 accounting for 90–95 % [12].

The recommendations for the treatment of type 1 diabetes *mellitus* in Portugal propose different insulin therapeutic regimens [13], which incorporate the scientific evidence available for insulin therapy. The recommendations for the treatment of type 2 diabetes *mellitus* propose different therapeutic regimens. In Portugal, the main documents on this matter are the recommendations of the Portuguese Society of Diabetology (*SPD*) [14–16] and the Clinical Guidelines of the Portuguese General Directorate of Health (*DGS*) [17–19], which incorporate the scientific evidence available for a wide range of antidiabetic drugs. The general approach to treat a patient with type 2 diabetes *mellitus* is the following: metformin is the first-line treatment; when a patient is not adequately controlled with metformin, a second noninsulin medication may be added; this second medication may be a sulphonylurea, a dipeptidyl peptidase-4 (DPP-4) inhibitor or a glitazone and, in the more recent guidelines, a glucagon-like peptide-1 (GLP-1) analog or a sodium-glucose cotransporter 2 (SGLT2) inhibitor. If even with this combined medication, the patient is not glycemic controlled, it is possible to add a third antidiabetic drug from other classes of noninsulin drugs. However, in every stage, if the patient presents a high level of glycated hemoglobin (> 10 %) [17, 19], therapy with insulin should be started.

Insulin is the only treatment available for type 1 diabetes *mellitus*, but due to the low prevalence of this disease, we can consider that consumption fluctuations for this drug are essentially related to type 2 diabetes *mellitus*. The consumption of noninsulin medicines is related to the treatment of type 2 diabetes *mellitus*.

Drug utilization studies in diabetes concerning the consumption of antidiabetic medicines often do not differentiate between the types of diabetes. Drug utilization studies on the consumption of medicines are usually expressed as prevalence, or exposition, of those drugs in a population. It can be the population of a country (e.g., Denmark [20]) or a region or other specific area (e.g., Granada metropolitan area of Andalusia region, Spain [21]).

In addition to analyzing the consumption of antidiabetic drugs, these studies provide insights into the effects of successive therapeutic guidelines and the introduction of new therapeutic classes of antidiabetic drugs into the market in clinical practice. The results can also provide valuable information for the rational use of medicines and allow decision-making regarding the policy for medicines.

The aim of this study was to characterize antidiabetic medicine consumption between 2005 and 2017, to evaluate the trends of these medicines in mainland Portugal, and to compare district consumption. An additional objective was to perform statistical analysis of the consumption of these drugs in different regions of Portugal.

## Methods

This study was observational, descriptive, and longitudinal; the setting was mainland Portugal (excluding Azores and Madeira).

All medicines, including antidiabetic medicines, were categorized according to the fifth level code of the Anatomical Therapeutic Chemical Classification System (ATC). For each ATC, there is a respective defined daily dose (DDD). All DDD units were reported in accordance with the ATC/DDD guidelines for implementation in 2018 [22]. Knowing the total units of DDD of a medicine for each year and each district, we obtained the consumption of that medicine for all 18 Portuguese districts from 2005 to 2017.

If the DDD units were for a specific district and a respective year, it was necessary to divide the DDD units by the annual average resident population in that district [23] and by the number of days depending on the specific year, considering the leap years. To express the DDD per thousand inhabitants per day (TID), it is necessary to multiply this value by 1000.

Consequently, it is possible to calculate the DDD/TID of mainland Portugal for a specific medicine using the total DDD consumption in all districts and the total resident population using the data of all districts.

From the fifth to the fourth second level of the ATC code, the DDD/TID ratios were calculated consecutively from sum totals of the respective DDD values for the group of drugs belonging to the fourth level of the ATC code for the district population and respective year. The same procedure was applied to each group of drugs for the third ATC level, in accordance with their categorization in the group of insulins and their analogs or to the group of noninsulin antidiabetic drugs. Finally, for the second ATC level, the DDD/TID was calculated for antidiabetic drugs.

The main outcome measure was the DDD/TID for mainland Portugal (including for all districts), between 2005 and 2017, for antidiabetic medicines, for insulins and analogs (including the different types of insulins), and for noninsulin medicines (including the different types of noninsulin medicines).

**Fig. 1:**
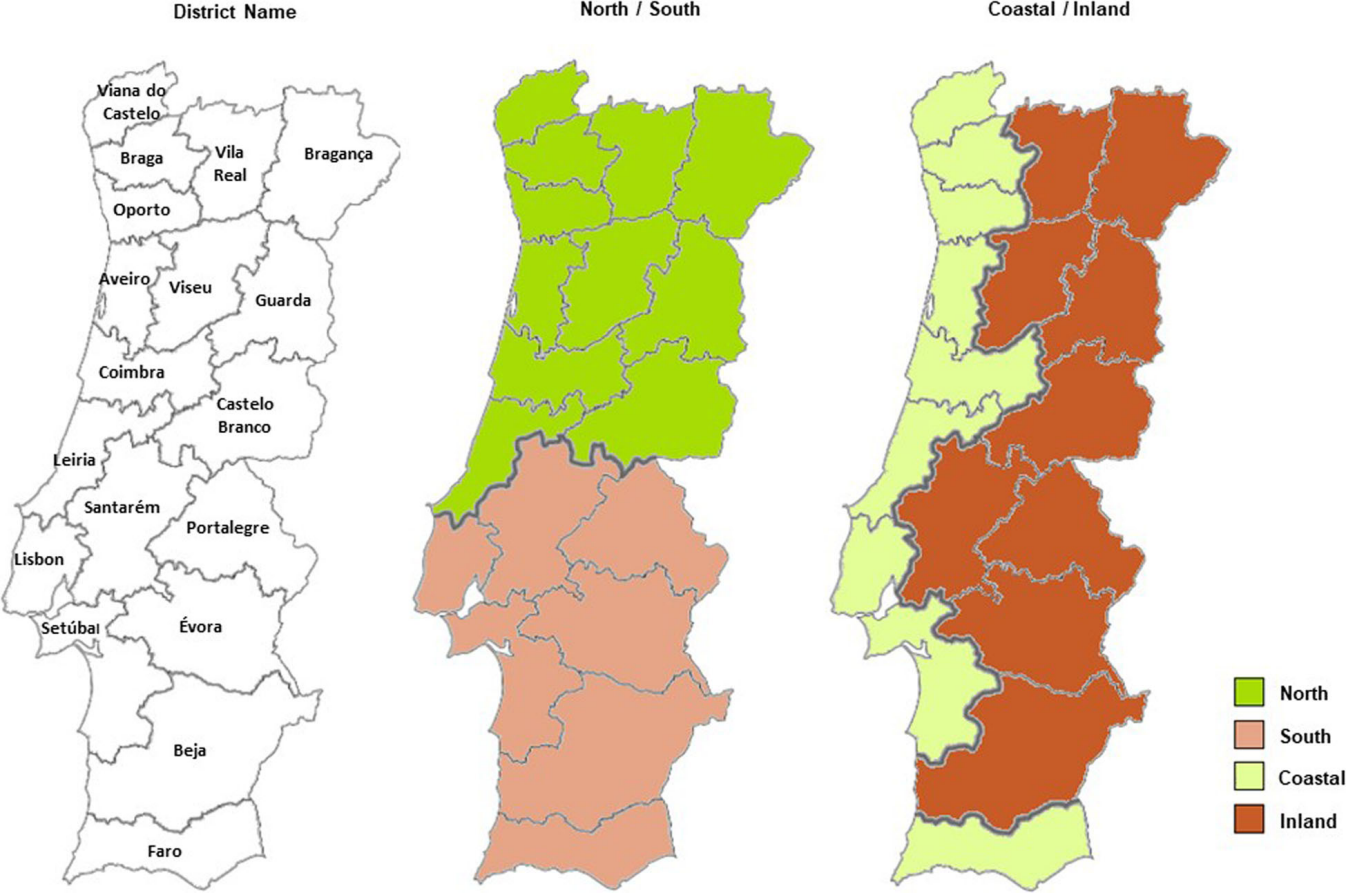
District names and groups categorized into north/south and coastal/inland classifications

The average levels of consumption of antidiabetic drugs, insulins and their analogs, and noninsulin antidiabetic drugs were calculated using the method described below to compare geographical areas that were larger than districts. All the districts were grouped into two categories (see Fig. 1) to identify the group of districts from the north and south, in addition to the group of districts from the coastal and inland regions. The north group included the districts most representative of the North and Central Health Regions, and the south group included the districts most representative of the Lisbon and Tagus Valley, Alentejo and Algarve Health Regions. Therefore, two groups that jointly, contained all the districts of mainland Portugal were obtained. The coastal group included the districts along the Atlantic strip, except for the Beja district, as it is essentially inland; all the remaining districts, including the Beja district, were included in the inland group.

The database used for this study was provided by Infarmed (Portuguese Medicines Authority). The database was created as a result of the reconciliation of invoices sent by community pharmacies to the Health Service’s Central Management (*ACSS*) for subsidized drugs in mainland Portugal. All antidiabetic drugs in Portugal are subsidized by the National Health Service irrespective of their income as long as they are either Portuguese or residing in Portugal. Patients obtain medication at community pharmacies by presenting a physician prescription. Community pharmacies are spread all over mainland Portugal, covering all districts, therefore allowing patients easy access to antidiabetic medicines. ACSS is not responsible for the invoice reconciliation for the Azores and Madeira regions; therefore, the database only has data for mainland Portugal.

The database contained the total DDD units for each antidiabetic medicine, according to the fifth level code of the ATC for the respective district (18 districts) and the respective year (between 2005 and 2017). For the oral fixed-dose combination drugs, the database did not include the annual DDD units. Instead, it contained the number of tablets consumed, for each of these medicines, per district and year. This information allowed the calculation of the annual DDD units for each oral fixed-dose combination drug.

The arithmetic calculations were performed using *Microsoft Office Excel 2007* software and the statistical calculations, including the Mann-Whitney U test, were performed using *SPSS (v25)* software. The level of statistical significance was set to *p* < 0.05, with a 95 % confidence interval (CI).

## Results

Table 1 presents the consumption of antidiabetic drugs, insulins and their analogs, and noninsulin antidiabetic drugs, including all types of these medicines, reported in DDD/TID from 2005 to 2017.

**Table 1:** The consumption of antidiabetic drugs in DDD/TID, from the second ATC level to the fourth ATC level, between 2005 and 2017

ATC	Medicines	2005	2006	2007	2008	2009	2010	2011	2012	2013	2014	2015	2016	2017
		DDD/TID												
A10	Antidiabetics	49.3	52.3	55.4	60.8	64.6	67.8	70.2	73.6	78.9	82.2	84.4	86.0	88.2
A10A	Insulins and analogues	5.3	6.9	7.7	8.6	9.2	10.1	10.9	11.8	13.1	13.9	14.4	15.0	15.4
A10B	Non-insulin antidiabetics	44.0	45.4	47.7	52.2	55.4	57.7	59.3	61.7	65.8	68.3	69.9	71.0	72.9
A10A	Insulins and analogues													
A10AB	Fast-acting insulins	1.0	0.9	1.2	1.6	1.9	2.2	2.6	3.0	3.4	3.7	3.9	4.1	4.3
A10AC	Intermediate-acting insulins	3.0	3.1	3.5	3.5	3.1	3.0	2.7	2.5	2.4	2.4	2.3	2.1	2.0
A10AD	Combined-acting insulins	1.4	2.9	3.0	3.0	3.0	3.0	3.0	3.0	3.1	3.1	2.9	2.8	2.6
A10AE	Long-acting insulins	< 0.1	< 0.1	< 0.1	0.5	1.1	1.8	2.6	3.3	4.1	4.8	5.3	5.9	6.4
A10B	Non-insulin antidiabetics													
A10BA	Biguanides	12.8	13.5	14.9	17.4	18.0	17.7	18.0	19.2	20.9	22.1	22.6	23.0	23.1
A10BB	Sulphonylureas	24.9	23.7	23.2	22.3	21.0	19.7	18.5	18.8	19.2	18.8	17.8	16.7	15.8
A10AF	α-glucosidase inhibitors	3.4	3.3	3.2	3.0	2.7	2.4	2.1	1.9	1.7	1.5	1.2	1.0	0.8
A10BG	Glitazones	0.2	0.2	0.6	1.0	1.0	1.1	1.1	1.0	0.9	0.8	0.6	0.5	0.5
A10BH	DPP-4 inhibitors	-	-	> 0.1	2.4	3.6	3.8	4.1	4.4	4.9	5.6	6.1	6.5	6.8
A10BJ	GLP-1 analogues	-	-	-	-	-	-	-	-	-	0.3	0.8	1.0	1.2
A10BK	SGLT2 inhibitors	-	-	-	-	-	-	-	-	-	< 0.1	1.0	2.0	3.0
A10BX	Other non-insulin antidiabetics	0.3	0.5	0.6	0.6	0.6	0.5	0.5	0.4	0.4	0.3	0.3	0.2	0.2
A10BD	Oral fixed-dose combinations	2.4	4.1	5.1	5.3	8.5	12.4	15.0	16.0	17.9	18.9	19.5	20.3	21.4

Note: The results presented in Table 1 were rounded to the first decimal place. The authors performed all calculations with raw data. Therefore, calculating annual growth rates with Table 1 data may lead to slightly different results than those presented in the text of this article

The consumption of antidiabetic drugs increased from 49.3 DDD/TID in 2005 to 88.2 DDD/TID in 2017, indicating an annual average growth rate of 6.6 %.

Insulins and their analogs, with a consumption of 5.3 DDD/TID in 2005 and 15.4 DDD/TID in 2017 exhibited an annual average growth rate that was greater than the increase in the consumption of antidiabetic drugs. Ultimately, insulins and their analogs represented 17.4 % of the total consumption of antidiabetic drugs.

The consumption of noninsulin antidiabetic drugs was 44.0 DDD/TID in 2005 and increased to 72.9 DDD/TID in 2017. The increase in consumption slowed between 2014 and 2017, with an annual average growth rate of 2.2 %. If noninsulin antidiabetic drugs represented 89.2 % of the total antidiabetic drugs consumed in 2005, their representativity decreased to 82.6 % in 2017.

### Insulins and their analogs (ATC fourth level)

Between 2014 and 2017, the consumption of long-acting insulins exhibited an annual average increase of 11.7 %. Furthermore, fast-acting insulins, despite the less marked variation, followed the trend of long-acting insulins. In 2017, these two types of insulin represented 70.1 % of the total insulin consumed.

In 2014, the combined-acting insulins represented of 22.1 % of the total insulin consumption. However, in 2017, this percentage decreased to 16.9 %. The intermediate-acting insulins, which were the most frequently consumed type of insulin in 2005, with a representativity of 56.7 % of all insulins, displayed a decrease in consumption that was accompanied by an increase in the consumption of other insulins and displayed a relative consumption of 13.0 % in 2017.

### Noninsulin antidiabetic drugs (ATC fourth level)

From 2005 to 2017, the consumption of biguanides increased from 12.8 to 23.1 DDD/TID, achieving first place in this group of noninsulin drugs. From 2014 to 2017, the consumption of sulphonylureas decreased annually, on average by 5.3 %. In 2017, the value remained at 15.8 DDD/TID. In 2017, the consumption of DPP-4 inhibitors reached 6.8 DDD/TID, representing an annual average growth rate of 7.3 % between 2014 and 2017.

In 2017, α-glucosidase inhibitors, glitazones, and a group of other noninsulin antidiabetic drugs (nateglinide) were consumed at lower levels in descending order.

In 2017, the consumption of new medicine classes, glucagon-like peptide-1 (GLP-1) analogs and sodium-glucose cotransporter 2 (SGLT2) inhibitors, reached 1.2 and 3.0 DDD/TID, respectively.

### Oral fixed-dose combinations

**Fig. 2:**
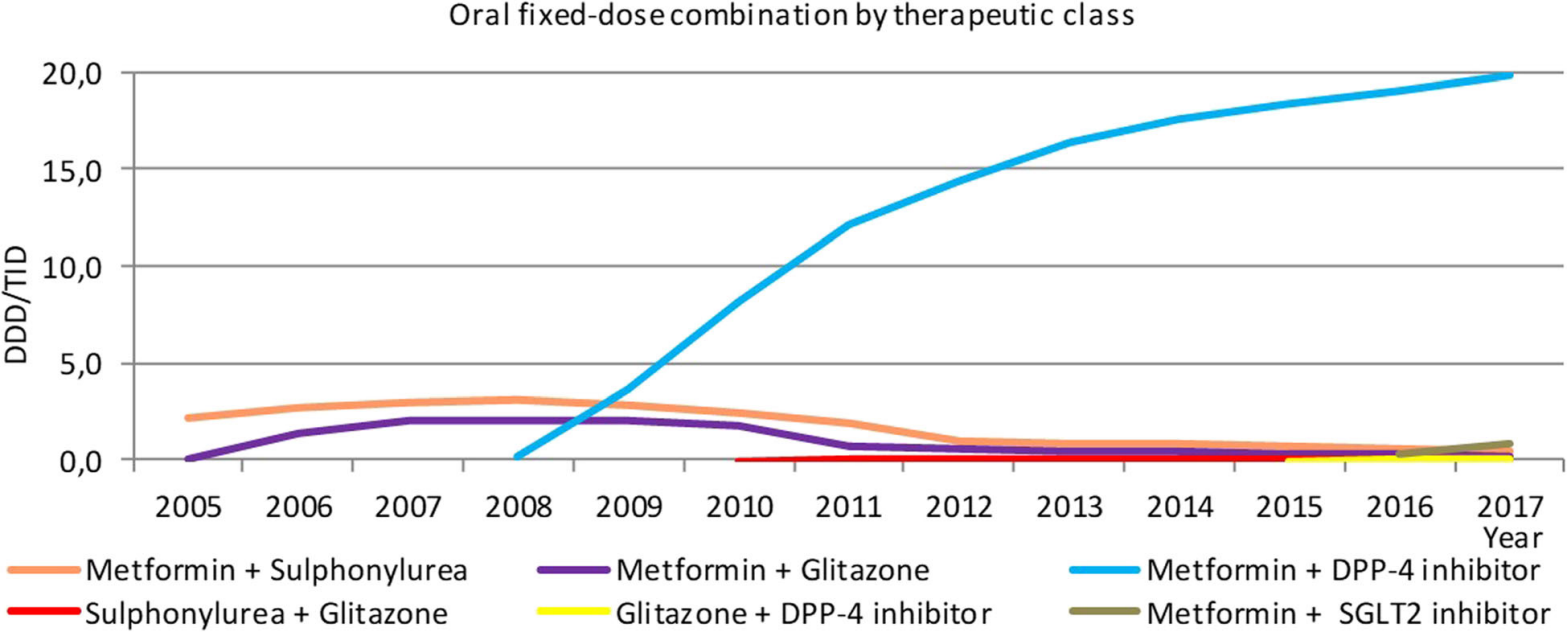
Consumption of oral fixed-dose combinations

Oral fixed-dose combinations experienced an annual average growth rate of 78.0 % between 2005 and 2014 and 4.5 % between 2014 and 2017. In 2017, these combinations achieved an impressive consumption level of 21.4 DDD/TID, which represents 29.4 % of noninsulin antidiabetic drugs (Fig. 2).

In 2005, oral fixed-dose combinations were practically represented by metformin combined with sulphonylurea, with a representativity of 95.2 %. However, with the decrease in the absolute level of consumption of this combination and the emergence and increased consumption of metformin combined with a DPP-4 inhibitor, the first combination represented 2.1 % and the second combination represented 92.6 % of the consumption of this drug class in 2017.

Notably, in 2017, the consumption of metformin and DPP-4 inhibitors reached 23.1 and 6.8 DDD/TID, respectively. However, in the same year, the consumption of metformin in combination with a DPP-4 inhibitor was 19.8 DDD/TID.

Among the new class of medicines, the consumption of metformin in combination with an SGLT2 inhibitor was 0.8 DDD/TID in 2017. This value achieved second place in the consumption of oral-fixed dose combinations.

### District-level variations

**Fig. 3:**
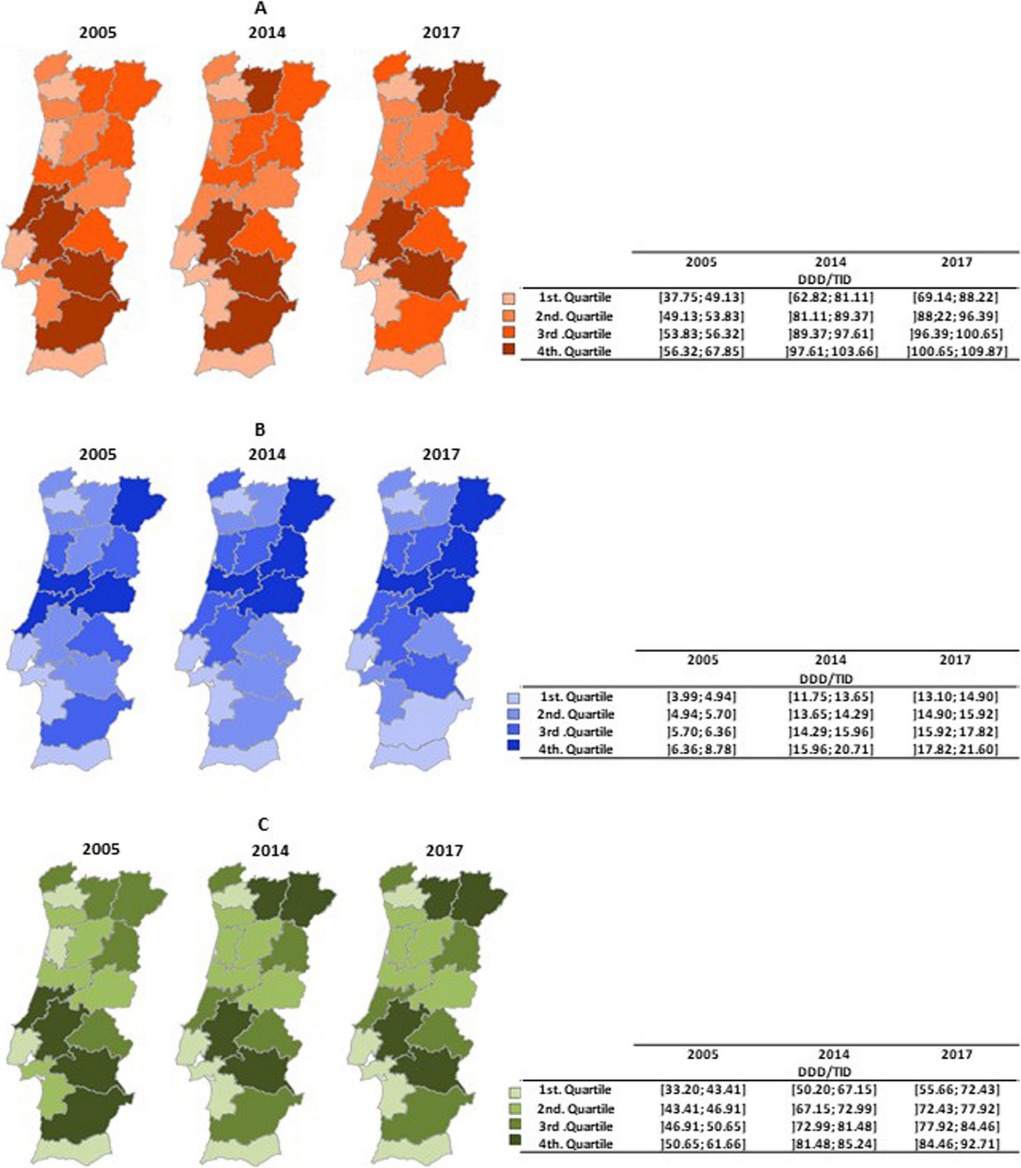
Consumption of drugs - **a** antidiabetics, **b** insulins and their analogs, and **c** noninsulin antidiabetics

In 2017, the districts with the highest levels of consumption of antidiabetic drugs were Bragança (109.9 DDD/TID) and Vila Real (107.8 DDD/TID), and the districts with the lowest levels of consumption were Faro (69.1 DDD/TID) and Lisbon (76.4 DDD/TID). From 2014 to 2017, the greatest increase in consumption occurred in the Bragança district, with an annual average increase of 4.2 %, and the lowest consumption was observed in Beja, with an annual average decrease of 1.8 % (Fig. 3: map A).

In 2017, the districts with the highest levels of consumption of insulins and their analogs were Coimbra (21.6 DDD/TID) and Castelo Branco (21.1 DDD/TID), and the districts with the lowest levels of consumption were Lisbon (13.1 DDD/TID) and Braga (13.3 DDD/TID). From 2014 to 2017, the greatest increase in consumption occurred in the Évora district, with an annual average increase of 4.8 %, and the lowest consumption occurred in Coimbra, with an annual average increase of 1.4 % (Fig. 3: map B).

In 2017, the districts with the highest levels of consumption of noninsulin antidiabetic drugs were Vila Real (92.7 DDD/TID) and Bragança (90.8 DDD/TID), and the districts with the lowest levels of consumption were Faro (55.7 DDD/TID) and Lisbon (63.3 DDD/TID). From 2014 to 2017, the greatest increase in consumption occurred in the Bragança and Viana do Castelo districts, both with an annual average increase in consumption of 3.7 %, and the lowest consumption was observed in Beja, with an annual average increase of 0.7 % (Fig. 3: map C).

### Northern/southern and coastal/inland district comparisons

The average consumption of antidiabetic drugs in the northern districts was 52.6, 89.3, and 96.9 DDD/TID in 2005, 2014, and 2017, respectively, and the values in the southern districts were 52.7, 86.9, and 90.2 DDD/TID in 2005, 2014, and 2017, respectively.

The average consumption of insulins and their analogs in the northern districts was 6.2, 15.8, and 17.3 DDD/TID in 2005, 2014, and 2017, respectively, and 5.3, 13.5, and 15.1 DDD/TID in the southern districts in 2005, 2014, and 2017, respectively.

The average consumption of noninsulin antidiabetic drugs in the northern districts was 46.4, 73.4, and 79.6 DDD/TID in 2005, 2014, and 2017, respectively, and 47.5, 71.4, and 75.2 DDD/TID in the southern districts in 2005, 2014, and 2017, respectively.

In the division between northern and southern districts, the difference between the average levels of consumption of antidiabetic drugs, insulins and their analogs, or noninsulin antidiabetics never achieved statistical significance in any year, except for insulins and their analogs in 2014 (Mann-Whitney U test: p = 0.020).

The average consumption of antidiabetic drugs in the coastal districts was 49.0, 80.6, and 87.3 DDD/TID in 2005, 2014, and 2017, respectively, and 56.3, 96.2, and 101.3 DDD/TID in the inland districts in 2005, 2014, and 2017, respectively. In each year analyzed, the average consumption of antidiabetic drugs in the coastal districts was always lower than that of the inland districts, indicating that these differences were statistically significant (Mann-Whitney U test: *p* = 0.019, *p* < 0.001, *p* = 0.001, respectively, for each year).

The average consumption of insulins and their analogs in the coastal districts was 5.5, 14.3, and 15.7 DDD/TID in 2005, 2014, and 2017, respectively, and 6.2, 15.5, and 17.1 DDD/TID in the inland districts in 2005, 2014 and 2017, respectively. In each year of observation, the average consumption of insulins and their analogs in the coastal districts was always lower than that in the inland districts. However, none of these differences were statistically significant.

The average consumption of noninsulin antidiabetic drugs in the coastal districts was 43.5, 66.7, and 71.6 DDD/TID in 2005, 2014, and 2017, respectively, and 50.1, 78.5, and 84.2 DDD/TID in the inland districts in 2005, 2014, and 2017, respectively. In each year analyzed, the average consumption of noninsulin antidiabetics in the coastal districts was always lower that in the inland districts, and these differences were statistically significant (Mann-Whitney U test: *p* = 0.014, *p* = 0.001, *p* = 0.003, respectively, for each year).

## Discussion

The data enable a multifaceted discussion, regardless of whether the results were obtained *per se* or whether they were attributed to any relationship with diabetes.

The analysis of the period between 2005 and 2017, representing 13 years of data, provides information on the overall trends and evolution of consumption for a reasonable period. However, due to the availability of new antidiabetic drugs and their inclusion in two guidelines published in 2013 and 2015 [15, 16], the period between 2014 and 2017 was highlighted in the results and the discussion.

In the period analyzed, 2005 to 2017, the consumption of antidiabetic drugs, insulins and their analogs, and noninsulin antidiabetic drugs was characterized by consistent growth, as shown in other studies [24, 25]. Notably, the proportion of consumption of insulins to noninsulin drugs also increased. The growth in the consumption of these medicines may be explained by an increasing number of people diagnosed with type 2 diabetes *mellitus* and by the fact that nonglycemic-controlled type 2 diabetes *mellitus* patients started being treated with an increasing number of noninsulin antidiabetic drugs or even accepting the use of insulin.

Consulting the OECD statistics for the total consumption of antidiabetic drugs reported in DDD/TID [26], per county, which includes data from 28 countries in 2017, Portugal is in 14th place in descending order (68.1 DDD/TID) between Belgium (71.0 DDD/TID) and South Korea (66.9 DDD/TID). Based on the results obtained from the present study (88.2 DDD/TID), Portugal would be the second country after Finland (92.1 DDD/TID) in terms of consumption. The likely explanation for the difference between the findings of this study and the OECD statistics is that the national authority database does not include the consumption of oral fixed-dose combination drugs in DDD, as explained in the methods section.

The consumption of insulins and their analogs increased substantially. From 2005 to 2014, their consumption increased at a high rate, and their consumption increased at a lower rate from 2014 to 2017. The sharp increase in the consumption of long-acting and, to a lesser extent, fast-acting insulins is very significant. In 2017, the situation was very different from the value of approximately 6 DDD/TID observed in 2000 [27]. The same trend has been observed in Denmark, where an increase in the number of users of insulins and their analogs, including fast-acting insulins, was observed from 1999 to 2014, and a substantial increase in the number of users of long-acting insulins was observed from 2004 to 2014 [20]. The same trend was observed in the Granada region of Spain in a study examining data collected from 2001 to 2014 [21].

At the end of the period analyzed here, the consumption of metformin exceeded the consumption of sulphonylureas, without considering the combination with a DPP-4 inhibitor. From a different perspective, a study performed in France revealed the same trend [28]. In 2006, 58 % of the patients initiating type 2 diabetes *mellitus* treatment with noninsulin antidiabetic drugs started with metformin as the first-line treatment, while 34 % initiated treatment with sulphonylureas. This difference increased significantly at the end of the study in 2013; 80 % started with metformin and 15 % were first with sulphonylureas.

The increase in the use of insulins and metformin is consistent with the clinical guidelines [14–19]. Of course, with some delay, these successive guidelines exerted a positive effect on the consumption of insulins and metformin over time. For example, a consistent decrease in sulphonylurea consumption and a consistent increase in metformin consumption were observed throughout the period studied, as metformin consumption only exceeded sulphonylurea consumption in 2012.

Surprisingly, the relative consumption of the fixed-dose combination of metformin with a DPP-4 inhibitor was higher than that of DPP-4 inhibitors alone. A relevant reference to the substantial consumption of DPP-4 inhibitors is the study by *C. Torre et al.* [29]. This study compares consumption in Portugal and the Netherlands based on data from 2013. In the Netherlands, DPP-4 inhibitors are less than 5 % of the total noninsulin antidiabetic drugs consumed, and fixed-dose combinations are consumed at residual levels. Based on our study, for the same year, the consumption of the fixed-dose combination of metformin with a DPP-4 inhibitor was 16.3 DDD/TID (corresponding to 24.8 % of noninsulin antidiabetic drugs), while DDP-4 inhibitor consumption was 4.9 DDD/TID (corresponding to 7.3 % of noninsulin antidiabetic drugs). Compared with the Netherlands, we concluded that Portugal exhibited substantial consumption of these drugs. Notably, the OECD reports an age-standardized diabetes prevalence of 4.6 % for the population aged 18–99 years in the Netherlands and 9.9 % in Portugal based on data collected in 2017 [10]. However, this last aspect does not explain the difference in consumption of this class of drug and its combination with metformin between the two countries. Most likely, these findings are related to differences in the clinical practice between Portugal and the Netherlands.

Returning to the topic of the consumption of a fixed-dose combination of metformin with a DPP-4 inhibitor and the high values observed in Portugal, the consumption of these drugs increased to 25.7 % in 2014 in relation to the total consumption of noninsulin antidiabetic drugs. In the same year, the value was 12.2 % for the Granada region [21]. Therefore, compared to this last study, these fixed-dose combination drugs are consumed at very high levels in Portugal.

Based on the data collected in the last few years, the consumption of the new medicine classes of GLP-1 analogs and SGLT2 inhibitors is expected to increase in the next few years.

The estimated prevalence of diabetes in 2015 was 13.3 %, and the estimated prevalence of diagnosed diabetes was 7.5 % [11]. Using the diagnosed population, an average daily consumption by individuals with diabetes of 1.12 DDD was obtained. We believe that this parameter is interesting, and a comparison of the consumption in different countries or regions would be useful, as this analysis would consider the population with diabetes instead of the total population.

Regarding the consumption of antidiabetic drugs, insulins and their analogs, or noninsulin drugs, at the district level, looking at the distribution per quartile, there were many changes in the positions on the years analyzed (2005, 2014, and 2017). In this way, it is interesting to observe, for each district, the dynamics for long-term (2005 *versus* 2014 and 2005 *versus* 2017) and medium-term (2014 *versus* 2017) relative changes in the consumption of these medicines.

Previous studies have observed differences in the consumption of antidiabetic drugs in different regions of Portugal [24, 25]. In Italy, substantial differences in the consumption of antidiabetic drugs were also observed at the regional level; higher levels of consumption were observed in the south that in the north [30].

When comparing the consumption of antidiabetic drugs and noninsulin drugs between north and south districts, the differences were not statistically significant. Concerning insulins and their analogs, although consumption in the northern districts was always higher than that in the southern districts, the results were statistically significant for only 2014. It is difficult to explain the reason for this last result, but it is probably related to several different factors.

Concerning the comparison of the consumption of insulins and their analogs between coastal and inland districts, there were no statistically significant differences in any of the years analyzed, but the average consumption was always higher in the inland districts. For the consumption of antidiabetic drugs and noninsulin drugs, the consumption was always higher in the inland districts, and these differences were statistically significant for all years under analysis (2005, 2014, and 2017). Inland districts have a larger elderly population, which probably explains the results obtained.

The concept of consumption in the market is not completely consistent with the concept of taking the drug. This study enabled us to characterize the levels of exposure of a population to a drug or groups of drugs, as well as their long-term fluctuations, and establish comparisons between these exposure levels in populations from different geographical areas. Nevertheless, we reasonably concluded that antidiabetic drugs are essentially consumed by people with diabetes, as other therapeutic indications and off-label uses are very rare. In this study, the limitations principally arise from the source of information. The data do not allow us to analyze the relationship between market consumption and the variables of the patient (its characterization, including HbA1c levels, years of diagnosed diabetes *mellitus* type 2, diabetes complications, etc.) or of the prescriber (prescriber characteristics such as age, medical specialty, type of medical institution, etc.).

The monetary values of the total outpatient drug market of 78.1 % in 2014 and 74.9 % in 2017 corresponded to the Health Service outpatient drug market [31]. However, all antidiabetic medicines are reimbursed by health services. Therefore, an important aspect to consider regarding the origin of the database is that the market value for insulin and noninsulin antidiabetic drugs reported in 2011 by the outpatient Health Service in relation to the total outpatient market value of these same drugs corresponded to 96.5 % [32]. This value reflects an approximately global representativity of the antidiabetic drugs in the outpatient market.

## Conclusions

The main results reveal a consistent increase in the consumption of antidiabetic drugs, insulins and their analogs, noninsulin drugs, long-acting and fast-acting insulins, metformin, DPP-4 inhibitors, and fixed-dose combinations of metformin with a DPP-4 inhibitor.

At the end of the period examined in the present study, the new classes of antidiabetic medicines, particularly SGLT2 inhibitors, achieved a representative consumption level.

In mainland Portugal, the regional differences in the consumption of antidiabetic drugs or their subtypes are marked by a geographical division between the coastal and inland districts rather than by a geographical division between the northern and southern districts. In this regional comparison, the consumption of several different drugs reached statistical significance.

The main contribution of this article is that it provides insights for future research analyzing the effects of changes in the consumption of antidiabetics on the treatment of the population with diabetes.

## Data Availability

All data are available at Infarmed, upon request.

## Abbreviations

*ACSS*: Health Service’s Central Management
ATC: Anatomical Therapeutic Chemical Classification System
DDD: Defined daily dose
*DGS*: Portuguese General Directorate of Health
DPP-4: Dipeptidyl peptidase-4
GLP-1: Glucagon-like peptide-1
Infarmed: Portuguese Medicines Authority
OECD: Organisation for Economic Cooperation and Development
*OND*: Portuguese National Diabetes Observatory
SGLT2: Sodium-glucose cotransporter 2
*SPD*: Portuguese Society of Diabetology
TID: 1000 inhabitants per day
WHO: World Health Organization
